# The impact of the COVID‐19 pandemic on radiotherapy delivery in Japan: An observational study based on the national database

**DOI:** 10.1002/cam4.6661

**Published:** 2023-10-30

**Authors:** Keisuke Tamari, Maiko Kishigami, Yasushi Nagata, Takashi Mizowaki, Takeshi Kodaira, Hiroshi Onishi, Kazuhiko Ogawa, Yoshiyuki Shioyama, Naoyuki Shigematsu, Takashi Uno

**Affiliations:** ^1^ Department of Radiation Oncology Osaka University Graduate School of Medicine Osaka Japan; ^2^ Department of Radiation Oncology Hiroshima University Hospital Hiroshima Japan; ^3^ Department of Radiation Oncology and Image‐Applied Therapy, Graduate School of Medicine Kyoto University Kyoto Japan; ^4^ Department of Radiation Oncology Aichi Cancer Center Hospital Aichi Japan; ^5^ Department of Radiology University of Yamanashi School of Medicine Yamanashi Japan; ^6^ Ion Beam Therapy Center SAGA‐HIMAT Foundation Saga Japan; ^7^ Department of Radiology Keio University School of Medicine Tokyo Japan; ^8^ Department of Radiology Chiba University Graduate School of Medicine Chiba Japan

**Keywords:** COVID‐19, hypofractionation, Japan, observational study, radiotherapy

## Abstract

**Background:**

This study analyzed the impact of the coronavirus disease 2019 (COVID‐19) pandemic on radiotherapy delivery in Japan using a high‐quality Japanese national database based on universal health coverage.

**Methods:**

We performed a retrospective observational study using National Database of Health Insurance Claims and Specific Health Checkups of Japan open data focused on radiotherapy between fiscal year (FY) 2019 and FY2020 and the number of COVID‐19 cases from the Ministry of Health, Labour, and Welfare. We statistically analyzed the relationship between the number of COVID‐19 cases and the number of radiotherapy deliveries in Japan as a whole and by prefecture.

**Results:**

The total number of external beam radiotherapy (EBRT) fractions was 4,472,140 in FY2019 and 4,227,673 in FY2020 (−5.8%). EBRT courses were 250,395 in FY2019 and 240,329 in FY2020 (−4.0%), stereotactic radiotherapy courses were 27,619 in FY2019 and 31,786 in FY2020 (+15.1%), and single‐fraction palliative radiotherapy courses were 4124 in FY2019 and 5255 in FY2020 (+21.5%). The total number of breast and prostate hypofractionated radiotherapy (HFRT) fractions was 155,773 and 48,188 in FY2019, and 200,256 and 84,230 in FY2020 (+28.6% and +74.8%), respectively. In the Pearson correlation analysis, EBRT fractions were lower, and breast HFRT fractions were higher in prefectures with more COVID‐19 cases.

**Conclusions:**

Overall, radiotherapy delivery in Japan was relatively stable after the pandemic, with an increase in HFRT. Also, EBRT fractions decreased, and breast HFRT were more likely to be used in prefectures with more COVID‐19 cases.

## Graphical abstract

Radiotherapy delivery in Japan was relatively stable after the pandemic. Hypofractionated radiotherapy (HFRT) fractions increased. Prefectures with more COVID‐19 cases had less external beam radiotherapy and more breast HFRT fractions.

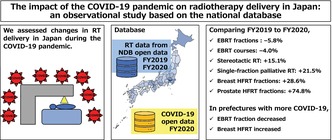

## 1 BACKGROUND

Coronavirus disease 2019 (COVID‐19) has negatively affected healthcare systems.^1^, ^2^ Cancer management has been also notably impaired by COVID‐19 worldwide such as delay in diagnosis, reduction in number of diagnosis, treatment distruption, and treatment delay.^3^ Our previous studies, using questionnaires administered to radiation oncologists in Japan, showed that COVID‐19 affected daily radiotherapy services. According to these studies, nearly all radiotherapy departments have continued to treat patients during the COVID‐19 pandemic in Japan. Some radiotherapy departments have experienced a decrease in patient numbers and have actively adopted hypofractionated radiotherapy (HFRT) over time.^4^, ^5^ A questionnaire‐based study from other countries suggested a decrease in the number of patients with COVID‐19.^6^, ^7^ The limitation of these studies, including ours, was that they did not directly demonstrate whether a reduction in the number of radiotherapy cases or promotion of HFRT was associated with the COVID‐19 pandemic.

Since universal health coverage was achieved in 1961, all Japanese people have easily accessed health services.^8^ The Ministry of Health, Labour, and Welfare compiles National Database of Health Insurance Claims and Specific Health Checkups of Japan (NDB). The NDB has become an extremely valuable data source for assessing the actual state of medical care in the Japanese population in almost total numbers. The NDB was released for research purposes in 2011. Several NDBs have been increasingly used in medical fields such as health economics, pharmacoepidemiology, clinical epidemiology, and other types of observational studies.^9^ However, to our knowledge, no studies have been conducted on radiotherapy using the NDB.

Since Japan had a complete count of patients with COVID‐19 from the beginning of the pandemic until September 2022, we could access accurate data on the number of patients with COVID‐19. We hypothesized that the precise changes in radiotherapy deliveries during the COVID‐19 pandemic in Japan could be tracked by analyzing the NDB and COVID‐19 databases.

This study aimed to determine how the COVID‐19 pandemic has changed the number of radiotherapy deliveries in Japan based on NDB open data and the COVID‐19 database.

## 2 METHODS

### 2.1 Data collection

We performed a retrospective observational study using NDB open data and COVID‐19 data. NDB open data are compiled by fiscal year (FY) and cover the period from April to March of the following year. From the Ministry of Health, Labour, and Welfare website, we downloaded the NDB open data focusing on radiotherapy between April 1, 2019, and March 31, 2020^10^, ^11^ and the daily number of patients with COVID‐19 from January 1, 2020, to March 31, 2021.^12^ Population data for each prefecture were downloaded from the 2020 census results as of October 1, 2020, from the e‐Stat website, a national database.^13^ This 2020 census data were used to calculate COVID‐19 incidence rates and the number of radiotherapy procedures performed by prefecture in 2019 and 2020.

This study was conducted in accordance with the tenets of the Declaration of Helsinki. No ethics committee review was required for this study because these data were public data with completely anonymized personal information.

### 2.2 Extraction of radiotherapy data

We analyzed radiotherapy data from NDB open data between April 1, 2019, and March 31, 2021. We counted external beam radiotherapy (EBRT) fractions based on the number of claims for radiotherapy fees per fraction, and the number of EBRT courses based on the number of claims for radiotherapy administration fees. EBRT courses were counted on the date when radiotherapy begins. The number of EBRT courses reflects the number of patients who received EBRT. Similarly, we counted stereotactic radiotherapy (SRT) courses based on the number of claims for SRT fees and single‐fraction palliative radiotherapy (SFPRT) fees. We counted the number of claims for an extra fee added to the reimbursement for each prostate and breast cancer fraction with a single dose of ≥2.5 Gy to evaluate HFRT for breast and prostate. The specific codes used for counting the number of fractions and courses in EBRT and HFRT are shown in Table S1. To assess how the COVID‐19 pandemic impacted HFRT delivery, we evaluated the number of SRT and SFPRT courses, as well as the number of HFRT sessions for breast and prostate, as indicators.

### 2.3 Statistical analysis

Prism 9 software (GraphPad Software, Boston, MA, USA) was used for statistical analysis. We performed Pearson's correlation analysis to confirm whether the number of COVID‐19 patients correlated with changes in radiotherapy delivery by prefecture. In correlation analysis, a p‐value of <0.05 was considered statistically significant, and a correlation coefficient *r* ≥ 0.3 or ≤−0.3 was considered statistically correlated.

## 3 RESULTS

### 3.1 Comparison of monthly EBRT between FY2019 and FY2020

**FIGURE 1:**
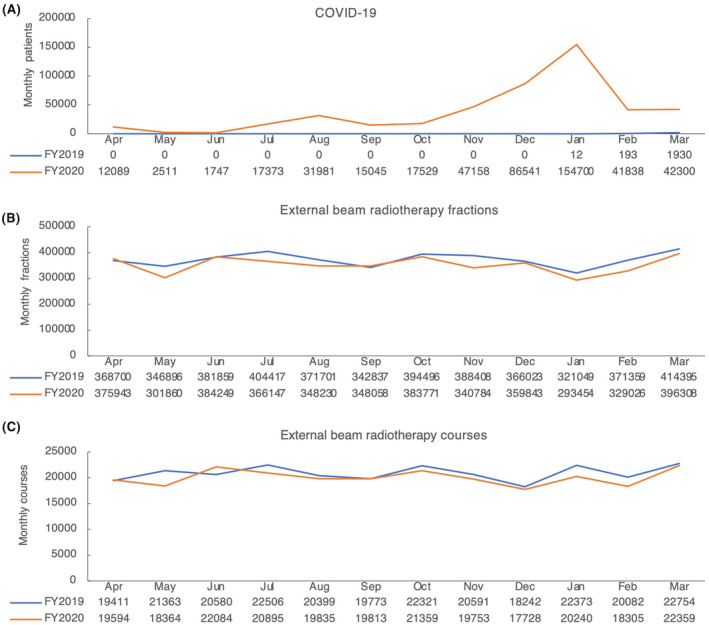
Monthly COVID‐19 cases and the number of external beam radiotherapy (EBRT) between 2019 and 2020. (A) The number of monthly COVID‐19 cases in FY2019 (blue) and FY2020 (red). (B, C) The number of monthly EBRT fractions and courses in FY2019 (blue) and FY2020 (red).

The total number of EBRT fractions was 4,472,140 in FY2019 and 4,227,673 in FY2020 (a 5.8% decrease). Also, the total number of EBRT courses was 250,395 in FY2019 and 240,329 in FY2020 (a 4.0% decrease). There was a notable initial decrease in the number of EBRT fractions and courses in May 2020, a month after the first wave of the pandemic in Japan. Afterward, in most months, the number of EBRT fractions and courses was below that in FY2019 (Figure 1A–C). These results indicate a decrease in EBRT in Japan after the COVID‐19 pandemic and a decrease in the number of patients with cancer receiving radiotherapy.

### 3.2 Comparison of monthly HFRT between FY2019 and FY2020

**FIGURE 2:**
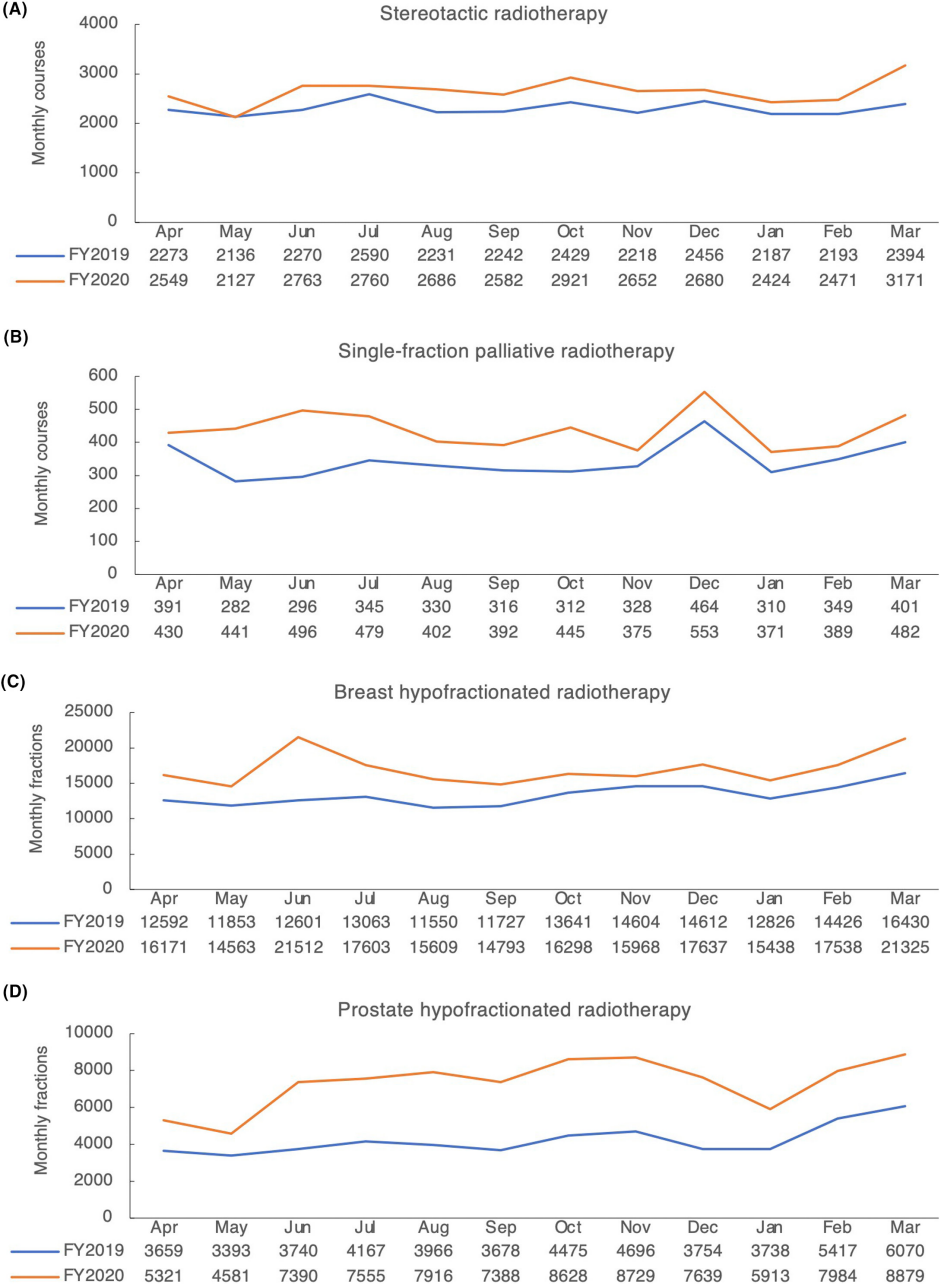
Monthly stereotactic radiotherapy, single‐fraction palliative radiotherapy, breast hypofractionated radiotherapy, and prostate hypofractionated radiotherapy between 2019 and 2020. (A) The number of monthly stereotactic radiotherapy courses in FY2019 (blue) and FY2020 (red). (B) The number of monthly single‐fraction palliative radiotherapy courses in FY2019 (blue) and FY2020 (red). (C, D) The number of monthly breast and prostate hypofractionated radiotherapy fractions in FY2019 (blue) and FY2020 (red).

Data on HFRT from the NDB included the number of SRT courses, SFPRT courses, breast HFRT fractions, and prostate HFRT fractions. Therefore, we compared these numbers by month for FY2019 and FY2020. The total number of SRT courses was 27,619 in FY2019 and 31,786 in FY2020 (+15.1% increase). There were more SRT courses in FY2020 than in FY2019 for most months. However, the number was slightly lower in May 2020, a month after the first wave of the pandemic in Japan (Figure 2A). The total number of SFPRT courses was 4124 in FY2019 and 5255 in FY2020 (+21.5% increase); it was higher in FY2020 than in FY2019 for all months (Figure 2B). The total numbers of breast and prostate HFRT fractions were 155,773 and 48,188 in FY2019, and 200,256 and 84,230 in FY2020 (+28.6% and +74.8% increase), respectively. The number of breast and prostate HFRT fractions was higher in FY2020 than in FY2019 in all months (Figure 2C,D).

These results show a consistent increase in HFRT fractions in Japan after the COVID‐19 pandemic, with a particularly notable increase in prostate cancer incidence compared to FY2019.

### 3.3 Correlation of the number of patients with COVID‐19 and changes in radiotherapy delivery by prefecture

**FIGURE 3:**
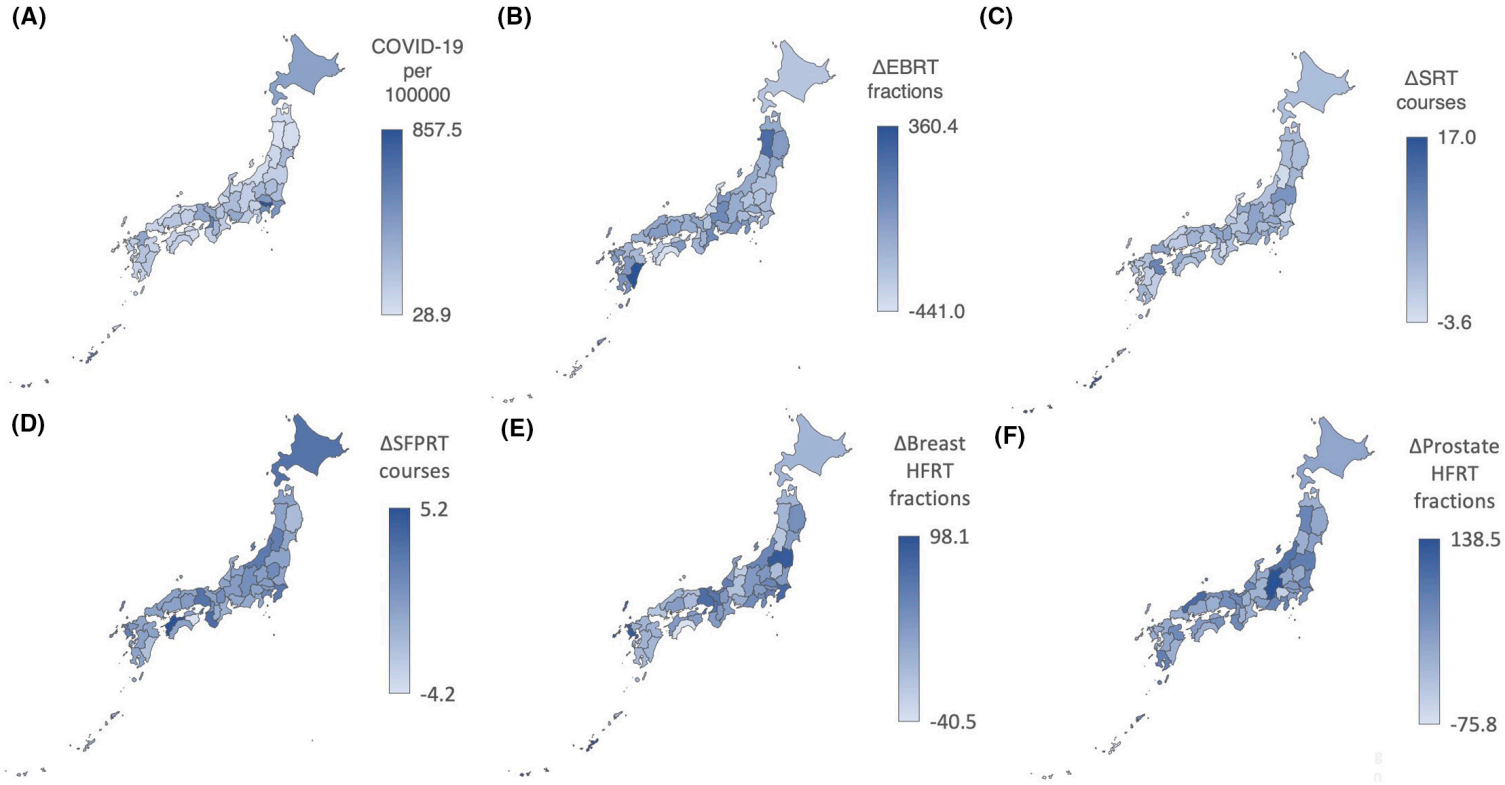
Annual COVID‐19 cases and the differences in the number of radiotherapy delivery by prefecture between 2019 and 2020. (A) Annual COVID‐19 cases per 100,000 population by prefecture. (B–F) The changes in external beam radiotherapy (ΔEBRT) fractions, stereotactic radiotherapy (ΔSRT) courses, single‐fraction palliative radiotherapy (ΔSFPRT) courses, breast hypofractionated radiotherapy (ΔBreast HFRT) fractions, and prostate hypofractionated radiotherapy (ΔProstate HFRT) fractions were indicated in the color of each prefecture on the map of Japan. The greater color intensity represents the greater value.

As described above, there are fewer EBRT fractions and courses and more HFRT fractions due to COVID‐19 in Japan. However, the prefectural differences are not known. The number of patients with COVID‐19 differed greatly by prefecture. The three prefectures with the highest number of patients with COVID‐19 per 100,000 people were Tokyo (857.5), Okinawa (645.7), and Osaka (588.2). Meanwhile, the three prefectures with the lowest numbers were Akita (28.9), Shimane (42.9), and Tottori 47.0 (Figure 3A). Therefore, we examined whether the prefectures with more COVID‐19 cases experienced greater changes in radiotherapy delivery.

The median difference between FY2019 and FY2020 in the number of EBRT fractions per 100,000 population (ΔEBRT fractions) was −148.1 (range, −441.0 to +350.4). The three prefectures with the highest ΔEBRT fractions were Miyazaki (+360.4), Akita (+210.4), and Gifu (+55.1), whereas the three with the lowest were Ishikawa (−441.0), Tokyo (−440.5), and Kochi (−432.7) (Figure 3B). The median difference between FY2019 and FY2020 in the number of EBRT courses per 100,000 population (ΔEBRT courses) was +10.8 (range, −18.4 to +40.4). The three prefectures with the highest ΔEBRT courses were Yamanashi (+40.4), Toyama (+35.6), and Gifu (+33.8), and the three prefectures with the lowest number of courses were Kochi (−18.4), Yamagata (−14.9), and Kagawa (−14.7) (Table S2).

The median difference between FY2019 and FY2020 in the number of SRT courses per 100,000 population (ΔSRT courses) was +1.8 (range, −3.6 to +16.9). The three prefectures with the highest number of ΔSRT courses were Okinawa (+17.0), Oita (+9.7), and Fukushima (+7.5), and the three with the lowest were Ishikawa (−3.6), Yamagata (−2.8), and Ibaraki (−2.7) (Figure 3C). The median difference between FY2019 and FY2020 in the number of SFPRT courses per 100,000 population (ΔSFPRT courses) was 0 (range, −4.2 to +5.2). The three most common prefectures were Ehime (+5.2), Wakayama (+3.5), and Hokkaido (+3.1), and the three with the lowest were Tokushima (−4.2), Miyazaki (−2.0), and Iwate (−1.7) (Figure 3D). The median difference between FY2019 and FY2020 in the number of breast HFRT fractions per 100,000 (ΔBreast EBRT fractions) was +27.0 (range, −40.5 to 98.1). The three prefectures with the highest numbers of ΔBreast EBRT fractions were Okinawa (+98.1), Nagasaki (+91.1), and Fukushima (+90.3), while the three with the lowest numbers were Kochi (−40.5), Toyama (−18.9), and Yamagata (−14.9) (Figure 3E). The median difference between FY2019 and FY2020 in the number of prostate HFRT fractions per 100,000 (ΔProstate EBRT fractions) was +15.4 (range, −75.8 to +138.5). The three prefectures with the highest numbers were Nagano (+138.5), Shimane (+99.5), and Niigata (+88.6), and the three with the lowest numbers were Okinawa (−75.8), Yamanashi (−50.9), and Gifu (−5.1) (Figure 3F). The numbers of COVID‐19 cases and radiotherapy deliveries by prefecture in FY2020 are shown in Table S2.

**FIGURE 4:**
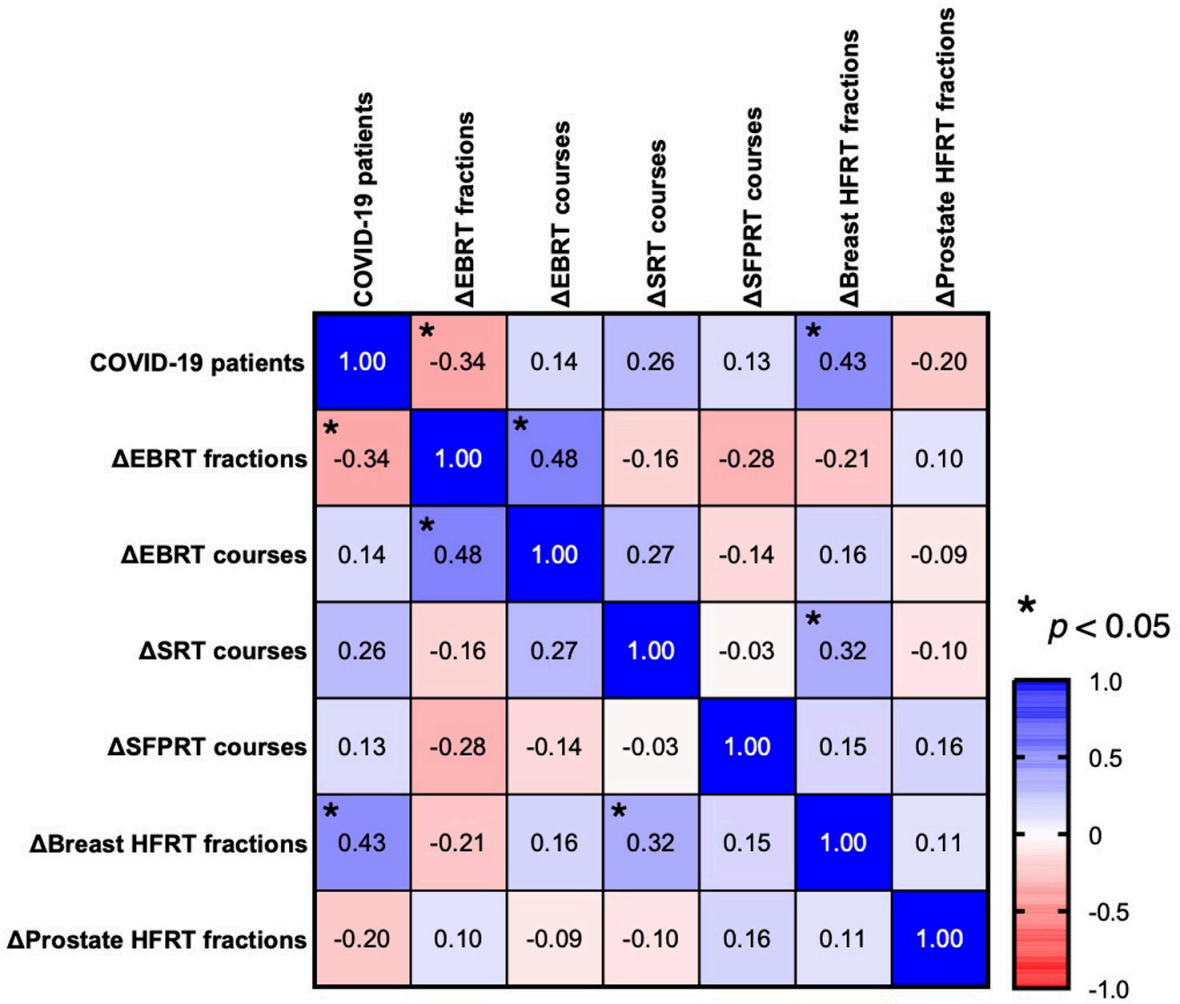
Pearson correlation analysis on the relationship between the number of patients with COVID‐19 and the difference in radiotherapy delivery per 100,000 population by prefecture. The correlation coefficient *r* corresponding to each cell is shown. The colors of the cells are shown in the lower right corner; blue indicates a positive correlation, and red indicates a negative correlation. *p‐*values < 0.05 are indicated by asterisks (*) in the upper‐left corner of the cell.

Pearson correlation analysis was performed to determine whether the number of patients with COVID‐19 per 100,000 by prefecture correlated with ΔEBRT fractions, ΔEBRT courses, ΔSRT courses, ΔSFPRT courses, ΔBreast EBRT fractions, and ΔProstate EBRT fractions by prefecture (Figure 4). The number of COVID‐19 cases per 100,000 per year by prefecture was negatively correlated with ΔEBRT fractions (*r* = −0.34, *p* < 0.05) and positively correlated with ΔBreast EBRT fractions (*r* = 0.43, *p* < 0.05). These results showed that prefectures in which COVID‐19 was significantly more prevalent had lower EBRT and higher breast HFRT fractions.

## 4 DISCUSSION

We used a high‐quality national Japanese database to study the impact of radiotherapy delivery during the early stages of the pandemic. Most reports on the impact of COVID‐19 on radiotherapy are questionnaire‐based,^6^, ^7^, ^14^, ^15^, ^16^ similar to our previous reports.^4^, ^5^ While they provided valuable information, one problem with these studies was that the response rate to the questionnaires was low, making it difficult to assess accurately the impact of COVID‐19 on radiotherapy. Accurate data are required to assess the impact of COVID‐19 on radiotherapy properly. Fortunately, we obtained the exact number of patients with COVID‐19 and radiotherapy treatments in Japan from a national database administered by the Ministry of Health, Labour, and Welfare.

In Japan, EBRT fractions and courses declined in May 2020 after the first wave of infection in April 2020. Although the number of patients with COVID‐19 was higher in the subsequent waves, the EBRT fractions and courses did not decline as much as in May 2020. A report analyzing the database of the largest radiotherapy institution in Scotland, which studied the same period as we did, showed a 10% decrease in the number of radiotherapy courses in FY2020 compared to FY2019.^17^ The decrease in EBRT courses in Japan was 4.0%, suggesting that radiotherapy activity was relatively stable during the early stages of the COVID‐19 pandemic.

Notably, increased use of SRT, SFPRT, and breast and prostate HFRT was also observed after the COVID‐19 pandemic, suggesting that many radiotherapy institutions have attempted to reduce patient infection risk and staff burden by reducing the number of hospital visits. However, there was no notable reduction in the overall EBRT fraction. We speculated that this was due to the small proportion of the HFRT fraction to the total fraction. The reasons radiotherapy delivery in Japan was not significantly impaired could be attributed to the fact that studies on COVID‐19 in radiotherapy departments were reported from around the world, which led to a better understanding of COVID‐19, and basic infection control measures were disseminated to both staff and patients. In addition, JASTRO's Ad Hoc Committee on COVID‐19 may have played a major role in maintaining radiotherapy delivery. This committee held free online educational seminars on COVID‐19 for radiotherapy staff nationwide,^18^ developed radiotherapy guidelines for the COVID‐19 pandemic,^19^ and monitored the nationwide status of radiotherapy delivery through periodic surveys to share information.^4^, ^5^ These efforts may have significantly impacted the maintenance of radiotherapy delivery in Japan. In addition, because several studies have shown that the proportion of cancers diagnosed at advanced stages is increasing during COVID‐19 pandemic,^7^, ^20^, ^21^ the numbers of SRT/SFPRT in palliative setting might have increased.

One unique aspect of the current study is evaluating the relationship between the number of patients with COVID‐19 per prefecture and radiotherapy delivery. The number of patients with COVID‐19 differed significantly between urban and rural areas. In the correlation analysis, EBRT fractions were lower, and breast HFRT fractions were higher in prefectures with more COVID‐19 cases. Notably, there was no significant correlation between the number of EBRT courses by prefecture and the number of COVID‐19 cases, suggesting that the number of patients receiving radiotherapy was not significantly affected by COVID‐19. The fact that prostate HFRT was not significantly correlated with the number of patients with COVID‐19 might be due to the Japanese health insurance system. Because prostate HFRT is permitted by the insurance system when we use the IMRT technique, HFRT of the prostate is unlikely to be adopted in Japan under the current situation, in which only nearly half of radiotherapy institutions (424 out of 794) provide IMRT in Japan.^22^ Therefore, if the IMRT provision system is insufficient in prefectures with many COVID‐19 patients, HFRT cannot be performed, so it is thought that the regional differences were not clear.

This study had several limitations. First, because the NDB open data were not tied to disease information, it was impossible to determine which cancer was treated with radiotherapy, except for breast and prostate HFRT. Therefore, it was impossible to compare them separately for each cancer, as in studies based in England and Scotland.^17^, ^23^ Second, not only the number of COVID‐19 cases but curfew restrictions due to emergency declarations and the recommendations for HFRT in JASTRO's COVID‐19 guidelines might promote HFRT in Japan. Third, the data may be slightly inaccurate owing to the aggregation rules of the NDB open data. In the NDB OpenData policy for disclosure, if the aggregate value is below 10, it is set to zero. This ensured that the individuals were not identified. In particular, some prefectures had SRT, prostate HFRT, and SFPRT scores of 0, but they may have been conducted with less than 10. Sensitivity analysis was performed, although results are not shown, and the results in Figure 4 showed that the policy of setting less than 10 to 0 had no problematic effect. Finally, the 2020 Census was dated October 1, 2020. We used 2020 Census data to calculate the 2019 and 2020 regional COVID‐19 incidence and radiotherapy delivery rates. The COVID‐19 incidence rate and radiotherapy delivery rates by prefecture are not accurate because we assumed the same population in 2019 and 2020. Despite these limitations, assessing radiotherapy delivery during the COVID‐19 pandemic using an accurate national database based on universal health coverage is worthwhile.

## 5 CONCLUSIONS

We assessed changes in radiotherapy delivery in Japan during the COVID‐19 pandemic using national databases for FY2019 and FY2020. Overall, radiotherapy delivery in Japan was relatively stable after the pandemic, with an increase in HFRT fractions. We also found that radiotherapy fractions decreased, and HFRT was more likely to be used in prefectures with more patients with COVID‐19. The long‐term impact of the COVID‐19 pandemic on radiotherapy delivery remains unknown and requires further study.

## AUTHOR CONTRIBUTIONS

**Keisuke Tamari:** Conceptualization (lead); formal analysis (equal); investigation (lead); methodology (lead); project administration (lead); writing – original draft (lead). **Maiko Kishigami:** Conceptualization (equal); data curation (lead); formal analysis (lead); methodology (equal). **Yasushi Nagata:** Conceptualization (equal); writing – review and editing (lead). **Takashi Mizowaki:** Supervision (equal). **Takeshi Kodaira:** Supervision (equal). **Hiroshi Onishi:** Supervision (equal). **Kazuhiko Ogawa:** Investigation (equal). **Yoshiyuki Shioyama:** Supervision (equal). **Naoyuki Shigematsu:** Supervision (equal). **Takashi Uno:** Methodology (equal); supervision (equal).

## FUNDING INFORMATION

This work was supported by JSPS KAKENHI Grant Number 23K07178.

## CONFLICT OF INTEREST STATEMENT

None.

## ETHIC STATEMENT

No ethics committee review was required for this study because these data were public data with completely anonymized personal information.

## Supporting information


Table S1.

Table S2.

## Data Availability

The data that support the findings of this study are openly available in sixth and seventh NDB open data at [https://www.mhlw.go.jp/stf/seisakunitsuite/bunya/0000177182.html], and in COVID‐19 database at [https://www.mhlw.go.jp/stf/covid‐19/open‐data_english.html].
